# Ofatumumab Treatment for Refractory Generalized Myasthenia Gravis Post‐Lung Adenocarcinoma Resection: A Case Report and Mechanistic Insight

**DOI:** 10.1002/iid3.70501

**Published:** 2026-08-19

**Authors:** Xia Long, Yanwen Xu, Juan Xiong, Wenfeng Peng, Jinhua Qiu, Yanxia Zhou

**Affiliations:** ^1^ Department of Neurology Shenzhen Second People's Hospital Shenzhen Guangdong China; ^2^ Department of Neurology The First Affiliated Hospital of Shenzhen University Shenzhen Guangdong China; ^3^ Shenzhen Institute of Translational Medicine Shenzhen Second People's Hospital Shenzhen Guangdong China; ^4^ School of Public Health Shenzhen University Shenzhen Guangdong China; ^5^ Department of Pathology Shenzhen Second People's Hospital, The First Affiliated Hospital of Shenzhen University Shenzhen Guangdong China; ^6^ Huizhou First Hospital The First Huizhou Affiliated Hospital of Guangdong Medical University Huizhou Guangdong China

**Keywords:** B‐cell depletion, lung adenocarcinoma, minimal manifestation status, ofatumumab, refractory myasthenia gravis

## Abstract

**Background:**

Myasthenia gravis (MG), an autoimmune neuromuscular disorder, may rarely coexist with malignancies such as lung cancer.

**Case Presentation:**

This case report presents the first documented use of ofatumumab, a fully human anti‐CD20 monoclonal antibody, in a 75‐year‐old female with acetylcholine receptor (AChR)‐antibody‐positive generalized MG (gMG) following lung adenocarcinoma resection. Initially diagnosed with ocular MG (MGFA Class I), the patient progressed to refractory gMG (MGFA Class IIIa) post‐thymectomy and tumor resection, unresponsive to corticosteroids, tacrolimus, and intravenous immunoglobulin. Due to persistent symptoms and steroid‐induced complications, subcutaneous ofatumumab (20 mg on Days 1, 7, and 14) was administered off‐label. Rapid clinical improvement was observed, with Myasthenia Gravis Composite (MGC) and MG Activities of Daily Living (MG‐ADL) scores declining from 20 to 10 and 17 to 10, respectively, within weeks. Minimal manifestation status (MMS) was sustained for 2 years with intermittent ofatumumab re‐administration (triggered by CD19/CD20 + B‐cell reconstitution > 1%), alongside no tumor recurrence or adverse events.

**Conclusion:**

This case highlights ofatumumab's potential efficacy and safety in managing refractory gMG post‐cancer resection, possibly via robust B‐cell depletion and reduced immunogenicity. These findings advocate for targeted B‐cell therapies as a viable strategy in complex, refractory MG cases, particularly where conventional immunosuppressants pose oncological concerns.

## Summary

This case highlights ofatumumab's potential efficacy and safety in managing refractory gMG post‐cancer resection, possibly via robust B‐cell depletion and reduced immunogenicity. These findings advocate for targeted B‐cell therapies as a viable strategy in complex, refractory MG cases, particularly where conventional immunosuppressants pose oncological concerns.

## 1 Introduction

Myasthenia gravis (MG), an autoimmune disorder targeting the neuromuscular junction, is primarily mediated by antibodies against the acetylcholine receptor (AChR) in approximately 85% of cases. While ocular MG (OMG) often progresses to generalized MG (gMG), concurrent malignancies such as lung cancer are rarely reported in these patients. Surgical interventions, infections, and inadequate immunotherapy are recognized triggers for MG exacerbation, yet the management of refractory gMG—defined by persistent symptoms despite steroids, intravenous immunoglobulin (IVIG), and conventional immunosuppressants—remains a significant clinical challenge. Notably, 15% of gMG patients fail to achieve minimal manifestation status (MMS), underscoring the need for novel therapeutic strategies [1].

B‐cell depletion therapies, particularly anti‐CD20 monoclonal antibodies (mAbs), have emerged as promising options for refractory MG [2, 3]. Rituximab, a chimeric anti‐CD20 mAb, demonstrates efficacy in depleting autoreactive B cells and short‐lived plasma cells, yet its utility in MG patients with malignancies remains underexplored [4, 5]. Ofatumumab, a fully human anti‐CD20 mAb, offers enhanced B‐cell lysis via dual mechanisms—antibody‐dependent cellular cytotoxicity (ADCC) and complement‐dependent cytotoxicity (CDC)—with reduced immunogenicity and infection risk [6]. Preclinical and clinical studies suggest anti‐CD20 agents may exert antitumor effects by modulating tumor‐associated immune microenvironments, though their role in cancer‐associated MG is unknown.

Here, we present the first case of an elderly patient with AChR‐positive gMG following lung adenocarcinoma resection, who achieved sustained MMS through intermittent of atumumab therapy. This report addresses critical gaps in understanding the interplay between MG exacerbation post‐thymectomy, the challenges of immunosuppression in cancer survivors, and the therapeutic potential of targeted B‐cell depletion in complex, refractory cases. By highlighting ofatumumab's rapid efficacy, safety profile, and compatibility with cancer surveillance, this study provides pivotal insights into optimizing immunotherapy for MG patients with coexisting malignancies.

## 2 Case Presentation

A 75‐year‐old female presented on October 11, 2022, with a 3‐month history of bilateral ptosis. Neurological evaluation revealed positive fatigue and neostigmine tests, while repetitive nerve stimulation (RNS) of facial and ulnar nerves showed no decrement. Serum anti‐AChR antibody were markedly elevated at 17.27 nmol/L (average value < 0.25 nmol/L), with negative anti‐muscle‐specific Tyrosine Kinase (anti‐MuSK) antibodies. Computed tomography (CT) revealed a mass in the lower right lung. Myasthenia Gravis Composite (MGC) and Myasthenia Gravis Activities of Daily Living (MG‐ADL) scores were 2/50 and 2/24. The patient was diagnosed with AChR antibody‐positive ocular MG (MGFA Class I). Comorbidities included undifferentiated connective tissue disease, hypertension, and impaired glucose tolerance.

Before surgery, her drooping eyelids returned to normal after treatment with pyridostigmine bromide (60 mg, three times daily). She did not receive immunosuppressive therapies such as corticosteroids, calcineurin inhibitors, or fast‐acting treatments (e.g., plasmapheresis, intravenous immunoglobulin, and intravenous methylprednisolone).

### 2.1 Surgical Intervention and Postoperative Course

**Figure 1:**
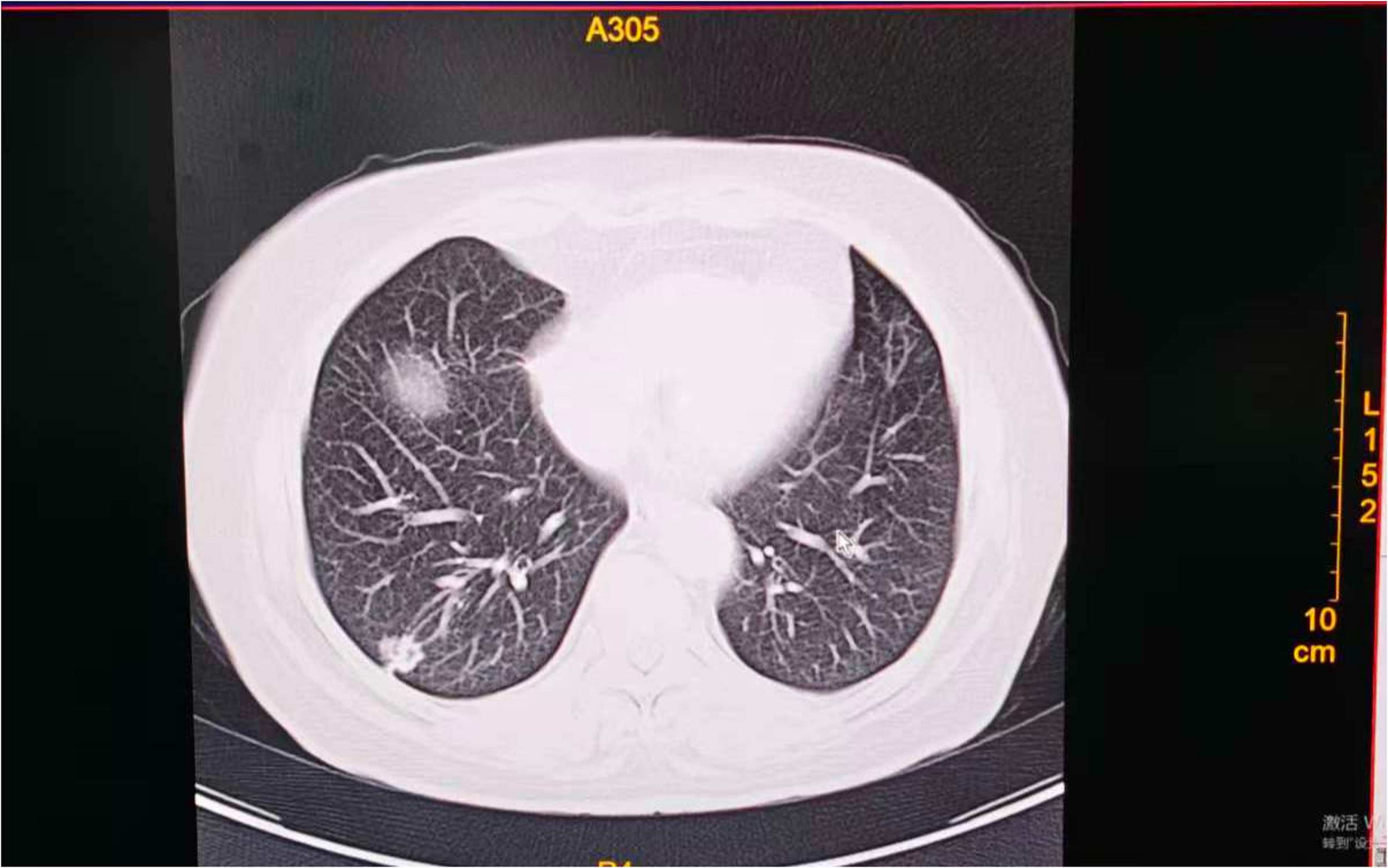
Results of Chest CT The image on the left shows the preoperative visible mass. The picture on the right is 6 months after surgery.

**Figure 2:**
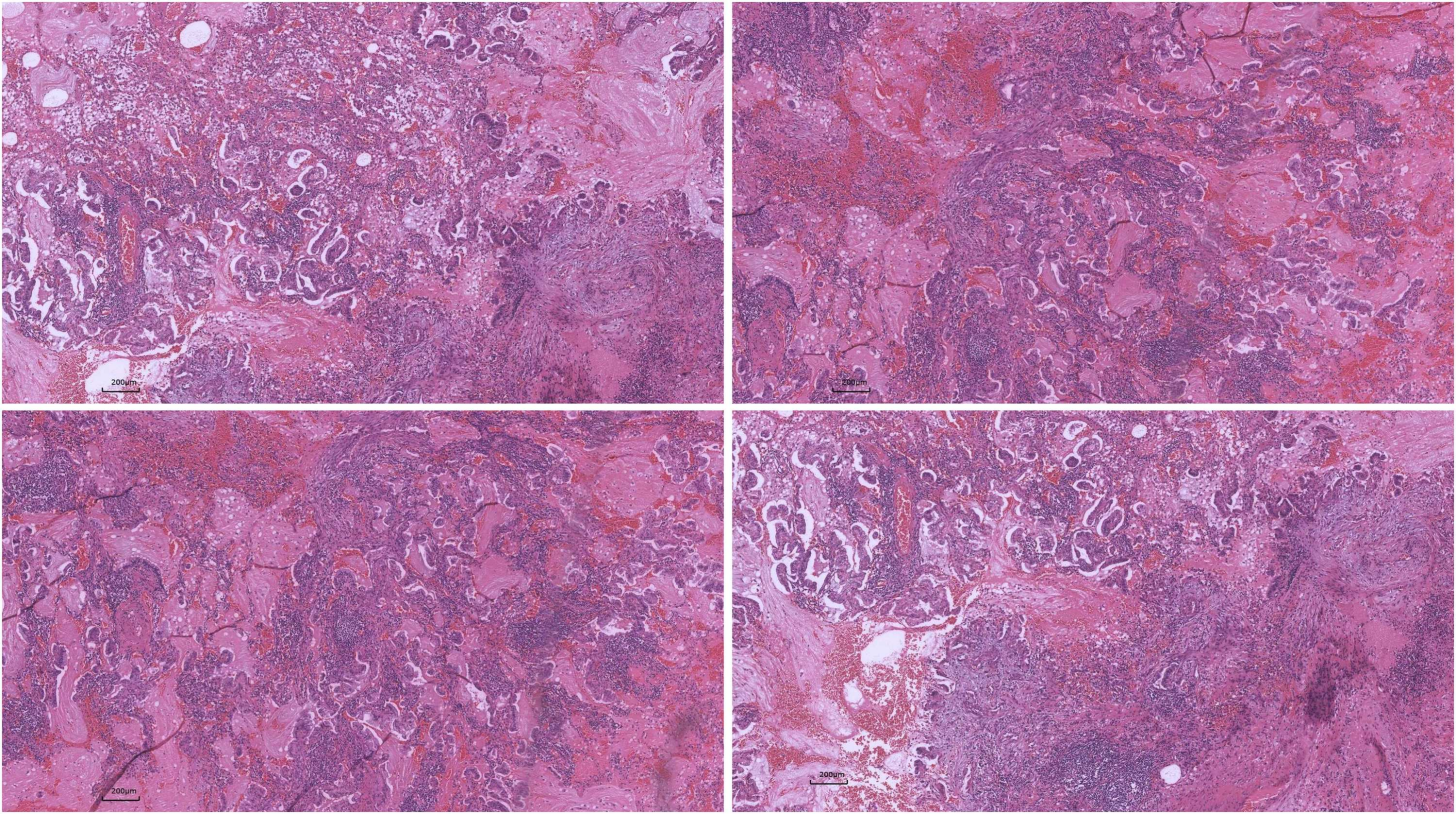
Results of histological examination: Histological grade: moderately differentiated, histological type: invasive adenocarcinoma. Mucous. Bronchial end: (−). Intravascular cancer thrombus, nerve invasion, and pleural infiltration were not involved and disseminated along the trachea (+). Parbronchial lymph nodes did not metastasize. Objective lens 10× Eyepiece 10× (HE staining) (hematoxylin‐eosin staining).

On November 27, 2022 (Figure 1), the patient underwent thoracoscopic partial resection of the right lung and anterior mediastinal enlargement of the thymus, and was diagnosed with right lower lung adenocarcinoma pT1bNoMO stage IA2 (Figure 2). Postoperative recovery was uneventful, with pyridostigmine maintained at 60 mg TID.

### 2.2 Disease Progression to Refractory Generalized MG

- By February 3, 2023, her symptoms showed progressive worsening. She experienced difficulty walking and swallowing, and experienced drooping eyelids and double vision. The patient was admitted to our hospital again. Neurological examination revealed bilateral ptosis with incomplete eyelid closure, Oculomotor nerver palsy with fatigable diplopia (duration:8 s at 45° gaze), Bulbar dysfunction (nasal speech, dysphagia), Respiratory involvement (FVC: 56% predicted), Neck flexor weakness (Medical Research Council [MRC] grade 0/5), marked limb girdle and axial weakness(she could sustain upper limb nd lower limb abduction at 90°for only 20 s, handgrip strength was reduced [6 kg, below normal for age/sex]). MGC and MG‐ADL scores escalated to 23/50 and 17/24, respectively, prompting reclassification as generalized MG (MGFA IIIa). The results of the laboratory examination revealed that ANA antibody, anti‐RO52 antibody, and anti‐mitochondrial antibody were positive. Hepatitis B DNA was negative. The patient refused RNS or single‐fiber EMG. Anti‐AChR is positive too, and anti‐P/Q VGCC antibodies are negative.

### 2.3 First‐Line Immunotherapy and Complications

The patient was hospitalized to start intravenous immunoglobulin (IVIg, 0.4 g/kg/qd for 5 days), methylprednisolone (20 mg qd), and pyridostigmine (60 mg QID). This regimen achieved transient stabilization of vital signs and partial symptom relief (Myasthenia Gravis Composite [MGC] score reduction from 23 to 20). However, she developed neuropsychiatric complications, including insomnia and depressive symptoms. Despite adjunctive therapies—trazodone (20 mg nightly) for sleep and duloxetine (20 mg daily) for mood stabilization—no significant clinical improvement was observed. Addition of tacrolimus (1 mg twice daily, trough level 4.2 ng/mL) as a steroid‐sparing agent failed to elicit clinical improvement. At the 1‐month follow‐up, the patient exhibited persistent axial weakness (Medical Research Council [MRC] neck flexion grade 0/5) and non‐ambulatory status, meeting diagnostic criteria for refractory generalized myasthenia gravis.

### 2.4 Ofatumumab Therapy and Outcomes

**Figure 3:**
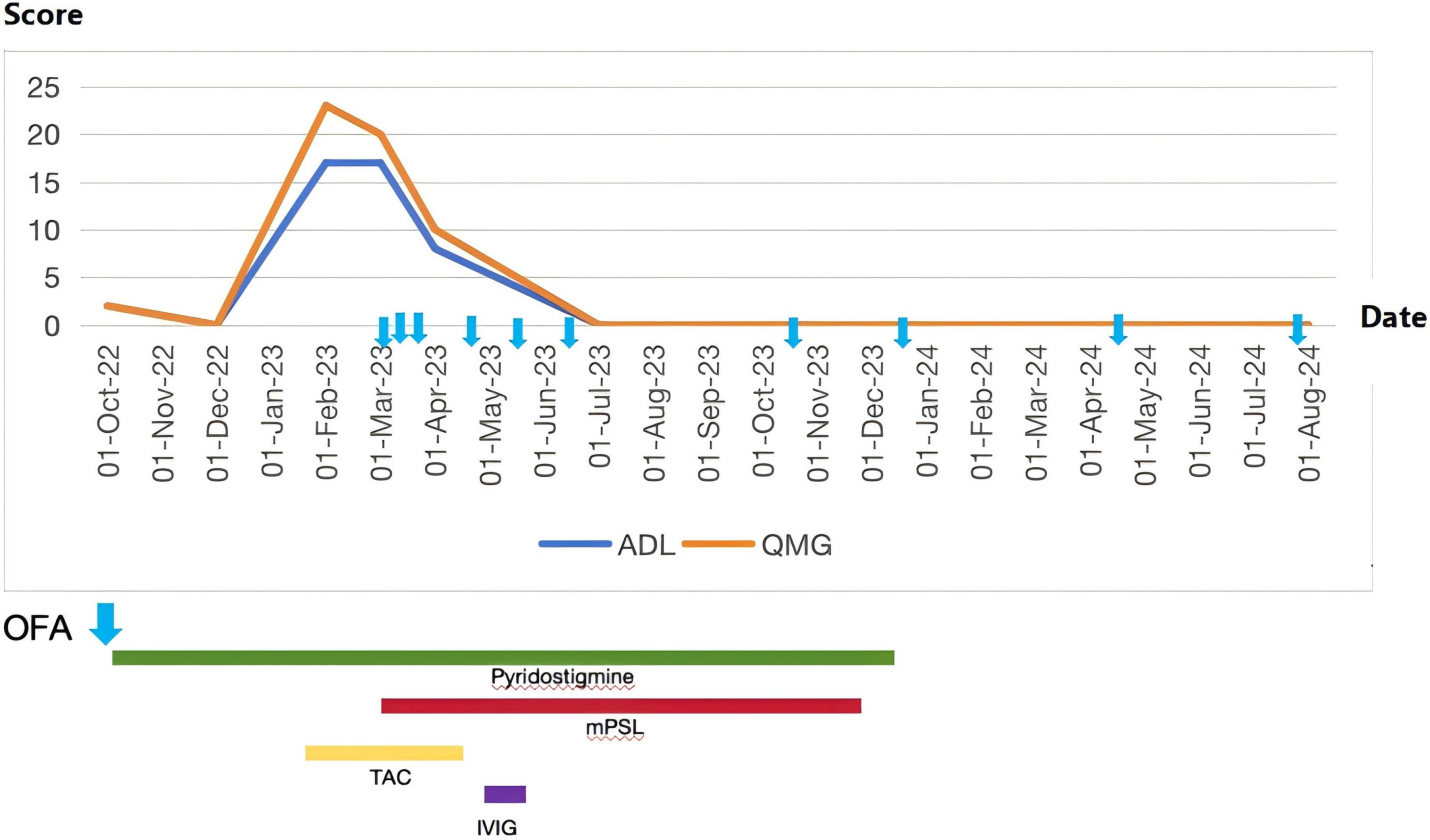
Specific medications and symptoms of the present patient.

Given the patient's refractory generalized myasthenia gravis, coupled with steroid‐induced neuropsychiatric complications and heightened oncological risks post‐lung adenocarcinoma resection, ofatumumab was selected as an off‐label therapeutic alternative. After obtaining ethics committee approval and informed consent, the patient received subcutaneous ofatumumab (20 mg on Days 1, 7, and 14), with concurrent tapering of tacrolimus to mitigate cumulative immunosuppression. Rapid clinical improvement was observed within days of the second dose: ptosis resolved, bulbar function normalized, and limb strength improved significantly (MRC neck flexion grade improved to 4/5, handgrip strength increased to 12 kg). Objective metrics reflected this progress: MGC and MG‐ADL scores declined from 20 to 10 and 17 to 10, respectively, within 2 weeks (Figure 3).

**Figure 4:**
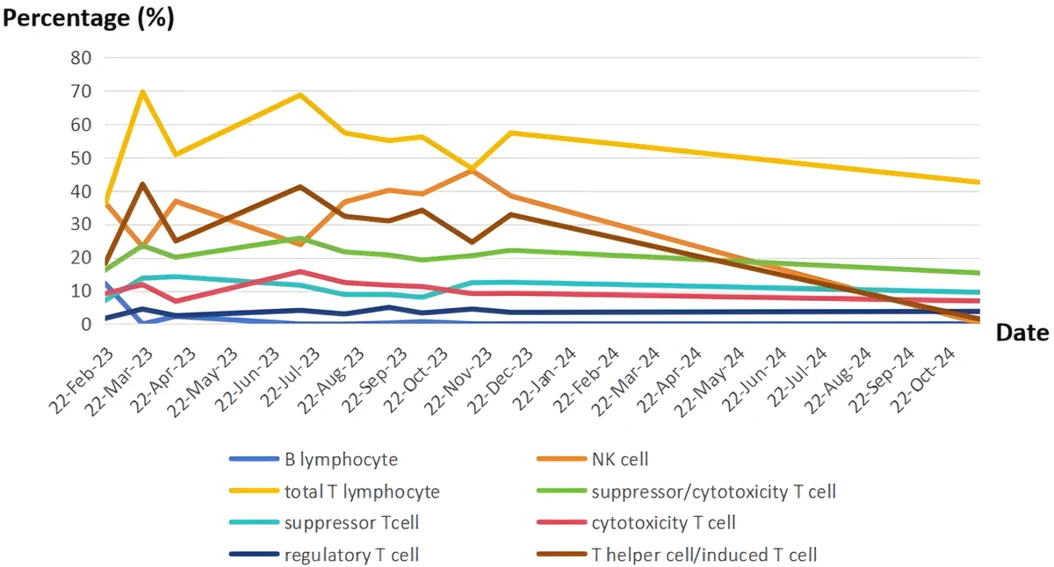
Fluctuating levels of different test results during ofatumumab use. IVIg, intravenous immunoglobulin; mPSL, methylprednisolone; OFA;QMG, myasthenia gravis quantitative score; MG‐ADL, myasthenia gravis daily Living Assessment Scale; TAC, tacrolimus.

B‐cell reconstitution (CD19^+^ /CD20^+^ > 1%) guided intermittent re‐dosing over 2 years, sustaining minimal manifestation status (MMS) without relapse. Notably, no adverse events—including infections or tumor recurrence—were observed, underscoring ofatumumab's safety in this high‐risk cohort. This tailored approach not only addressed refractory MG but also aligned with long‐term oncological surveillance priorities, offering a paradigm for managing overlapping autoimmune and malignant conditions. The clinical medications and symptom improvement of the patients are presented in Figure 3. Figure 4 shows the fluctuating levels of the test results during ofatumumab use.

## 3 Discussion

The association between myasthenia gravis (MG), thymic abnormalities (hyperplasia or neoplasia), and malignancies such as lung cancer has been increasingly recognized. Approximately 10‐15% of MG patients have thymoma, about 30% of thymoma patients are combined with MG, and about 70% of MG patients have thymic hyperplasia [7, 8, 9]; further, patients with Lambert‐Eaton Vasthenic syndrome (LEMS) as the initial symptom of lung cancer have also been reported [10]. In this patient, postoperative progression from ocular MG (MGFA Class I) to refractory generalized MG (gMG; MGFA Class IIIa) may be attributed to residual abnormal T cells persisting after thymectomy, which continue to drive autoantibody production [11, 12, 13]. Although thymectomy reduces anti‐AChR antibody levels, symptom exacerbation in some patients post‐surgery—particularly those with high antibody titers—highlights the potential role of postoperative immune microenvironment dysregulation in aggravating disease via both humoral and cellular pathways [8, 9]

Conventional immunosuppressants (e.g., tacrolimus, azathioprine) remain widely used for MG, but their delayed onset (3–4 months), oncological risks (especially with alkylating agents like cyclophosphamide), and steroid‐related complications (e.g., neuropsychiatric symptoms) limit their utility in post‐cancer patients [14, 15].

Recently, anti‐CD20 monoclonal antibodies (e.g., rituximab) targeting B cells have emerged as promising options for refractory MG by depleting short‐lived plasma cells and autoreactive B cells [2, 3]. Many studies and experience in clinical practice have confirmed that a decline in B‐cell depletion reduces antibody levels and relieves clinical symptoms in patients with MG. Rituximab can further deplete short‐lived plasma cells and improve symptoms in patients with refractory gMG. RTX functions through the ADCC pathway. In contrast, Ofatumumab, as a fully human anti‐CD20 monoclonal antibody, offers a lower immunogenicity profile compared to chimeric agents like rituximab, minimizing the risk of neutralizing antibody formation—a critical consideration in a patient with concurrent autoimmune and oncological comorbidities [16]. Second, its dual mechanism of B‐cell depletion—potent complement‐dependent cytotoxicity (CDC) and antibody‐dependent cellular cytotoxicity (ADCC)—enables robust depletion of autoreactive B cells and short‐lived plasma cells, which are central to AChR antibody production in refractory MG [17].

A previous study reported that RTX can deplete CD20‐positive B cells not only in primary lung tumor and lung‐associated lymph nodes, but also in the normal lung tissue in a patient with rheumatoid arthritis who underwent thoracic surgery for non‐small cell lung cancer. Several murine models have confirmed that targeted approaches for enhancing conventional B cells could effectively inhibit tumor growth and lung metastasis by stimulating B cell ligands, which could thereby increase the antitumor responses, including antibody‐specific recognition, lysis of tumor cells, reduction of the immunosuppressive environment, and/or tumoricidal‐cell response via the Fas/FasL pathway. Additionally, B cells can act as tumor suppressors in vivo, but not in all tumors. Longer term clinical observation is needed to determine its efficacy and safety.

This study presents the first reported use of ofatumumab in refractory gMG following lung cancer resection. Its efficacy may stem from: Potent B‐cell depletion: Rapid reduction of AChR antibody levels via CDC/CDC‐mediated clearance of CD20 + B cells and short‐lived plasma cells; Immune microenvironment modulation: Preclinical evidence suggests anti‐CD20 therapy may suppress tumor‐associated immunosuppressive signals (e.g., regulatory B‐cell activity), potentially lowering postoperative recurrence risk [18, 19]; Safety profile: Subcutaneous administration convenience, absence of steroid‐related complications, and no observed infections or tumor recurrence during long‐term follow‐up align with post‐cancer immune management needs.

Notably, an intermittent dosing strategy (retreatment triggered by CD19^+^/CD20^+^ B‐cell reconstitution > 1%) sustained symptom control (MMS) for 2 years, offering a novel framework for personalized therapy. However, long‐term safety concerns (e.g., secondary malignancies) require further validation. Current retiming criteria lack consensus; while the 6‐month B‐cell reconstitution interval recommended by the Swedish Multiple Sclerosis Society provides a reference [19, 20], dynamic immune monitoring (e.g., CD27^+^ memory B‐cell ratios) may optimize dosing schedules.

## 4 Conclusion

This study demonstrates, for the first time, that the fully human anti‐CD20 monoclonal antibody ofatumumab safely and effectively controls refractory gMG following lung cancer resection. Its efficacy correlates with profound B‐cell depletion, low immunogenicity, and potential antitumor effects. The intermittent dosing strategy achieved sustained remission (MMS for 2 years) without tumor recurrence or severe adverse events, underscoring its unique value in complex MG cases with malignancies. However, long‐term risks (e.g., immune deficiency or secondary tumors) necessitate large‐scale validation. Future multicenter randomized controlled trials should clarify indications, dosing protocols, and safety of ofatumumab in cancer‐associated MG, advancing targeted B‐cell therapies as a frontline option for these patients.

## 5 Limitations

This is a single case report without a control group, which limits generalizability. In this case report, the patient received multiple concomitant medications—including intravenous immunoglobulin, corticosteroids, pyridostigmine, and tacrolimus (with gradual tapering of the latter)—both before initiating ofatumumab and during the treatment period. Consequently, the independent efficacy of ofatumumab cannot be precisely delineated.

## Author Contributions

**Yanxia Zhou:** data curation, investigation, visualization, writing – original draft, writing – review and editing. **Xia Long:** data curation, investigation, methodology. **Yanwen Xu:** Validation, visualization. **Juan Xiong:** Data curation, investigation, methodology. **Wenfeng Peng:** formal analysis, visualization, validation; pathological slide evaluation, histological image quality control and pathological data interpretation for all figures. **Jinghua Qiu:** validation, visualization, writing – review and editing.

## Ethics Statement

This study was approved by the Ethics Committee of Shenzhen Second People's Hospital (approval number: 2024‐042‐01PJ). The patient provided written informed consent for publication.

## Conflicts of Interest

The authors declare no conflicts of interest.

## Data Availability

The data that support the findings of this study are available on request from the corresponding author. The data are not publicly available due to privacy or ethical restrictions.
